# Correction to “Exhaled breath condensate contains extracellular vesicles (EVs) that carry miRNA cargos of lung tissue origin that can be selectively purified and analyzed”

**DOI:** 10.1002/jev2.12453

**Published:** 2024-05-21

Megan I. Mitchell, Iddo Z. Ben‐Dov, Kenny Ye, Christina Liu, Miao Shi, Ali Sadoughi, Chirag Shah, Taha Siddiqui, Aham Okorozo, Martin Gutierrez, Rashmi Unawane, Lisa Biamonte, Kaushal Parikh, Simon Spivack, Olivier Loudig

In the originally published article, author Kaushal Parikh's name was misspelled. This has been corrected in the online version of the article.

We apologize for this error.

